# The Influence of *Cynips quercusfolii* on the Content of Biofunctional Plant Metabolites in Various Morphological Parts of *Quercus robur*

**DOI:** 10.3390/molecules30132687

**Published:** 2025-06-21

**Authors:** Anna Przybylska-Balcerek, Kinga Stuper-Szablewska

**Affiliations:** Department of Chemistry, Poznań University of Life Sciences, 60-637 Poznan, Poland; kinga.stuper@up.poznan.pl

**Keywords:** English oak, *Quercus robur*, galls, *Cynips quercusfolii*, oxidative stress antioxidant compounds, phenolic acids, flavonoids, tannins

## Abstract

English oak (*Quercus robur*) hosts over 200 species of galls formed by insect larvae, most notably the oak gall wasp (*Cynips quercusfolii*). These galls result from the abnormal growth of plant tissue in response to oviposition, acting as a shelter and nutrient source for the larvae. In addition, the galls trigger oxidative stress in the host plant, resulting in the increased production of reactive oxygen species (ROS). This stress response promotes the biosynthesis of antioxidant compounds, including phenolic acids, flavonoids, and tannins. To our knowledge, this is the first study to monitor seasonal changes in phenolic acids, flavonoids, and tannins in relation to *C. quercusfolii* infestation over a complete vegetation cycle using integrated UPLC profiling and statistical modeling PCA. For the first time, the contents of phenolic acids, flavonoids, and tannins were assessed throughout the vegetation cycle—from flowering to acorn fall. Results showed that galls affect the biochemical profile of the whole plant, suggesting a systemic response to local infection. The results provide new insights into oak defense responses and suggest that gall formation may be associated with systemic metabolic shifts potentially involved in stress mitigation. Furthermore, the study supports the further investigation of oak galls as a valuable source of polyphenols for pharmacological and industrial applications.

## 1. Introduction

**Figure 1:**
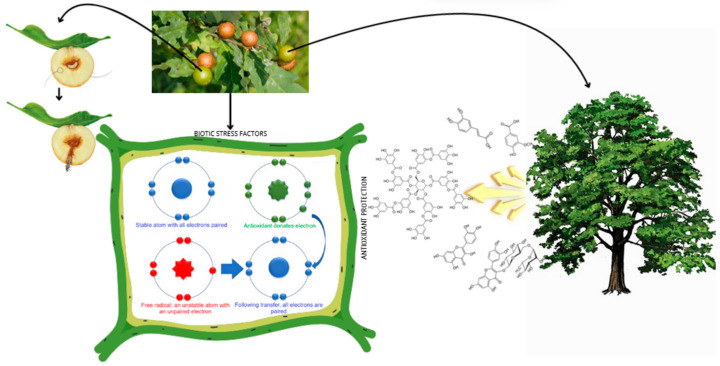
Biotic stress factors and antioxidant protection.

English oak (*Quercus robur*) from the beech family includes over 200 species that differ in morphology [1,2,3]. One of the interesting phenomena associated with English oaks is galls, which are formed on various morphological parts of the oak as a result of feeding insect larvae, including the larvae of the oak gall moth (*Cynips quercusfolii, C. quercusfolii*). These are abnormal growths of plant tissue formed on leaves, buds, flowers, and young branches due to stings and egg-laying by female *C. quercusfolii* [3,4,5]. They cause irritation, which leads to the overgrowth of regenerative tissue (callus). Galls act as a shelter and food source for larvae and are characterized by a high tannin content, which can constitute up to half of the dry weight of the gall. The presence of galls can contribute to increased oxidative stress [6], during which the host plant is exposed to the additional production of free radicals (reactive oxygen species, ROS). Physiologically, ROS are important signaling molecules leading to increased cell proliferation, differentiation, and maturation [7]. ROS plays an important role in immune responses and immunometabolism, during which the production of antioxidant compounds is intensified. Antioxidants include bioactive metabolites, including phenolic acids, flavonoids, and tannins (Figure 1). Therefore, historically, galls of *Quercus* sp. have been used as traditional remedies in the treatment of inflammatory conditions, including diarrhea and dysentery, abdominal pain, toothache, and tooth decay, as well as in postnatal care and in the fight against metabolic abnormalities and diseases related to oxidative stress [8,9,10]. Pharmacologically, *Quercus* sp. galls have antibacterial, antioxidant, anti-inflammatory, anticancer, antifungal, antiviral, antiprotozoal, antiamoebic, antiulcer, larvicidal, tooth and gum tonic, antidiabetic, cardioprotective, hepatoprotective, antiparkinsonian, antitumor, and wound healing properties [1,9,11,12,13,14,15]. Despite its numerous therapeutic properties, the long-term use of galls is not recommended as they may cause side effects such as irritation of the gastric mucosa, nausea, and vomiting [16]. In addition to its medicinal uses, the industrial use of *Quercus* sp. galls dates back to ancient times, with the galls being used for tanning leather, as a coloring agent for paintings, and as natural dyes for carpet yarns, respectively, as a component of ink. Galls of the *Quercus* sp. genus are characterized by a high tannin content (50–70%) [2,11]. In addition to tannins, the diverse phenolic profile mainly includes numerous flavonoids and simple phenolic compounds, such as phenolic acids, hydroxyphenols, and coumarins, and in smaller numbers, representatives of other groups of phenolic compounds, i.e., phenolic aldehydes, naphthodianthrones, acylphloroglucinols, phenolic alcohols, and stilbenes [17,18,19] However, their most valuable substance is gallic acid, which belongs to the group of phenolic acids. An analysis of the polyphenol content of plant-based raw materials is important from the point of view of searching for new sources of these compounds, qualitative assessments, and assessments of the level of oxidative stress to which the plant is exposed. Galls are an example of an interaction between an insect and a plant. So far, the literature indicates that the intensification of gallic acid biosynthesis is local [20,21,22]. Based on our own experiences and the immune mechanisms of other plants, it is known that even a local infection can affect the metabolism of the entire plant. Based on these premises and the fragmentary knowledge on this subject, we decided to conduct preliminary studies.

The aim of this study was to assess the oxidative stress response in *Quercus robur* L. (English oak) induced by larval infestation of the oak gall wasp (*Cynips quercusfolii*). The assessment was based on two parameters: (1) the antioxidant capacity of plant tissues, measured using the ABTS^+^ (2,2′-azino-bis(3-ethylbenzothiazoline-6-sulfonic acid) radical-scavenging assay, and (2) the profile of selected bioactive secondary metabolites known to be involved in plant defense responses to biotic stress. Quantitative chemical analyses were performed on four anatomical parts of the tree: leaves, oak sawdust, flowers, and fruits (acorns). Each tissue type was sampled from both infested and non-infested (control) oak specimens to compare the systemic impact of gall formation. The working hypothesis was that larval infestation by *C. quercusfolii* significantly alters the antioxidant status and secondary metabolite composition not only in the infected leaves but also in other parts of the tree. This assumes the existence of a systemic response to local biotic stress.

## 2. Results and Discussion

**Figure 2:**
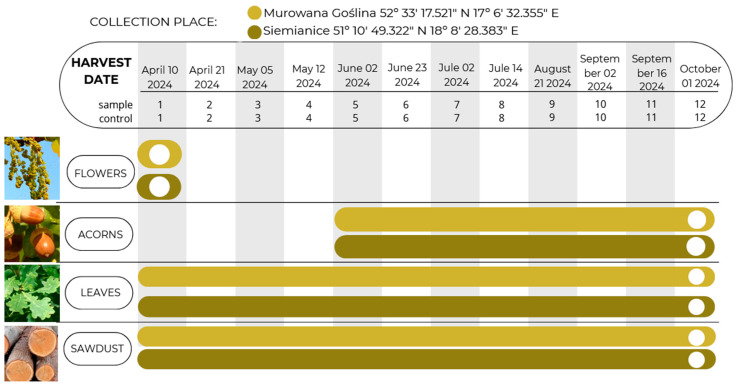
Date and place of collection of English oak samples (flowers, acorns, leaves, sawdust); sample, tree with galls; control, healthy tree.

During the study, chemical analyses were performed to determine the content of biofunctional metabolites in various anatomical parts of the English oak at two-week intervals from flowering to full acorn maturity (Figure 2).

**Figure 3:**
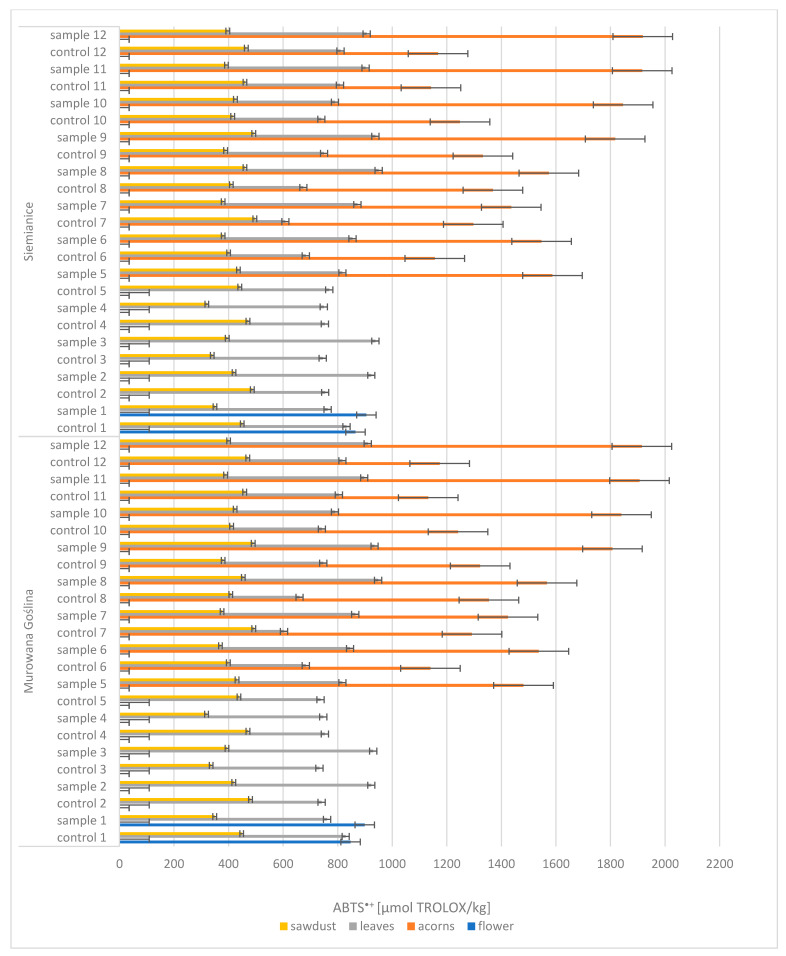
ABTS^+^ Murowana Goślina, and ABTS^+^ Siemianice; sample, tree with galls; control, healthy tree.

One of the analyzed parameters was antioxidant activity, expressed by ABTS^+^. Based on the obtained results, it was found that among the four morphological parts examined (i.e., flowers, acorns, leaves, and sawdust), acorns were characterized by the highest antioxidant activity (Figure 3). In the context of the analysis, the results for acorns from a healthy tree (CONTROL: 1132–1369 µmol TROLOX/kg) differed significantly from the results for acorns from an infected tree (SAMPLE: 1424–1918 µmol TROLOX/kg), with antioxidant activity increasing with acorn maturation. On the other hand, leaves and sawdust showed lower radical-scavenging activity ABTS^+^ concentrations, which did not change significantly during maturation. ABTS values for leaves were as follows: CONTROL, 603–829 µmol TROLOX/kg; SAMPLE, 747–950 µmol TROLOX/kg. For sawdust, they were as follows: CONTROL, 336–496 µmol TROLOX/kg; SAMPLE, 319–492 µmol TROLOX/kg.

In summary, acorns showed the highest antioxidant activity, and their ABTS^+^ concentration varied depending on the tree’s health and the maturation stage. Other parts of the plant, such as leaves and sawdust, were characterized by lower but stable values of this parameter, which implies no variation between gall-affected and control samples.

Based on the conducted studies, it was found that the content of phenolic compounds in the flowers of the English oak did not show significant differences depending on the location or between the variants infected with gall and those without galls.

**Figure 4:**
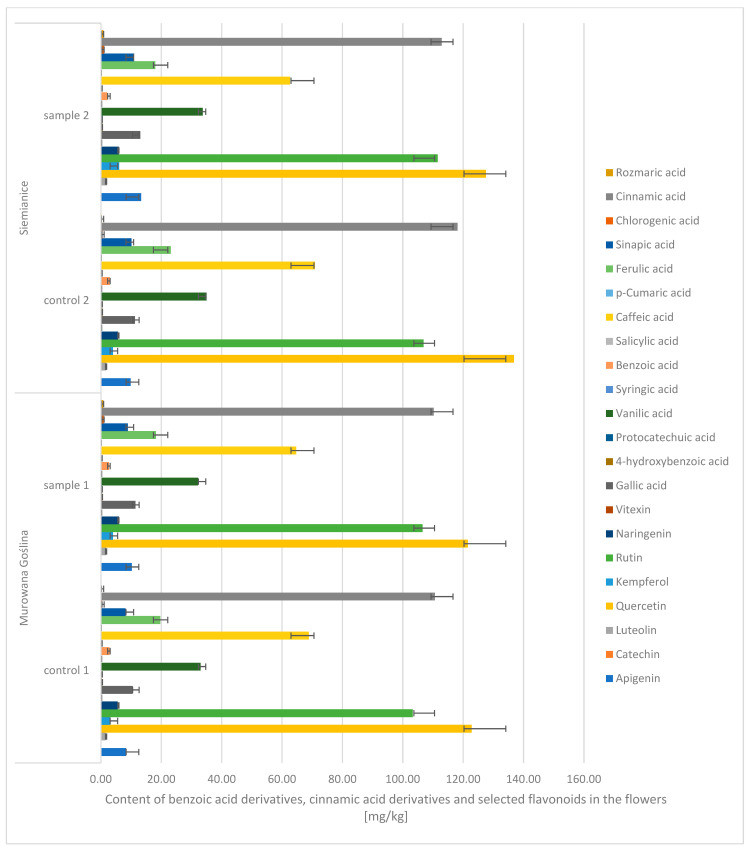
Contents of benzoic acid derivatives, cinnamic acid derivatives, and selected flavonoids in the flowers of the English oak; sample, tree with galls; control, healthy tree.

Due to the chemical structure and properties of phenolic compounds, they were divided into three main groups: benzoic acid derivatives, cinnamic acid derivatives, and flavonoids (Figure 4). Among benzoic acid derivatives, vanillic acid was found to have the highest content. Its content was 68–71%. In turn, among all cinnamic acid derivatives, the highest content was found for cinnamic acid. Its content was 53–55% (CONTROL: 110–118 mg/kg d.m. (dry mass); sample: 112–118 mg/kg d.m.), and the caffeic acid content was 31–33% (CONTROL: 68–70 mg/kg d.m.; P: 62–64 mg/kg d.m.). In turn, during the analysis of flavonoids, the highest content was noted: quercetin, i.e., 48–52% (CONTROL: 122–136 mg/kg d.m.; SAMPLE: 122–127 mg/kg d.m.), rutin, i.e., 40–43% (CONTROL: 103–106 mg/kg d.m.; SAMPLE: 106–111 mg/kg d.m.). The lowest values were recorded for the following: syringic acid (CONTROL: 0.13–0.23 mg/kg d.m.; SAMPLE: 0.16–0.24 mg/kg d.m.), *p*-coumaric acid (CONTROL: 0.09–0.11 mg/kg d.m.; SAMPLE: 0.11 mg/kg d.m.), catechins (CONTROL: 0.02–0.03 mg/kg d.m.; SAMPLE: 0.02–0.03 mg/kg d.m.), and vitexin (CONTROL: 0.13–0.18 mg/kg d.m.; SAMPLE: 0.14–0.24 mg/kg d.m.).

**Figure 5:**
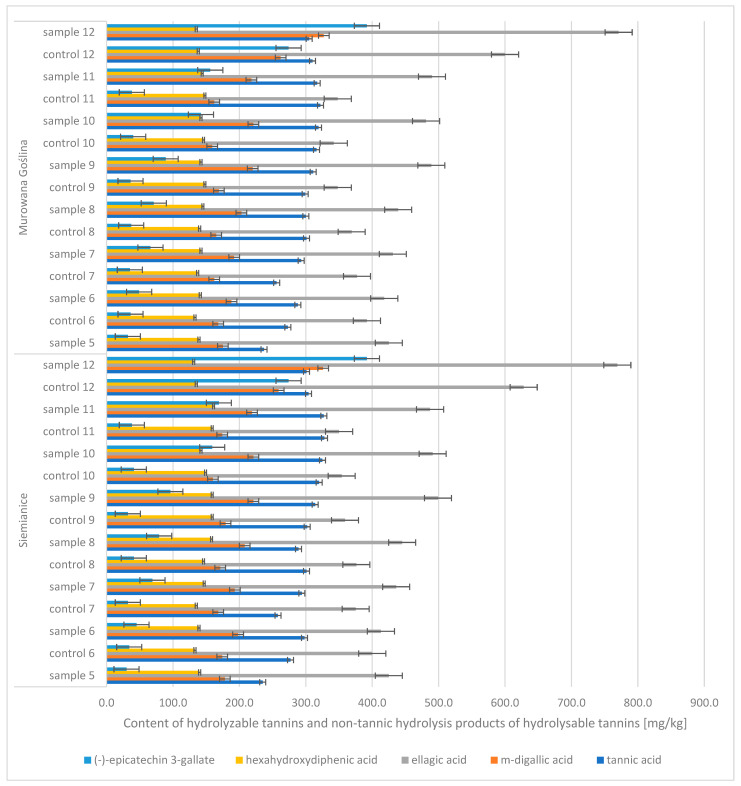
Contents of hydrolyzable tannins and non-tannic hydrolysis products of hydrolyzable tannins in English oak acorns (Murowana Goślina, Siemianice) (mg/kg); sample, tree with galls; control, healthy tree.

**Figure 6:**
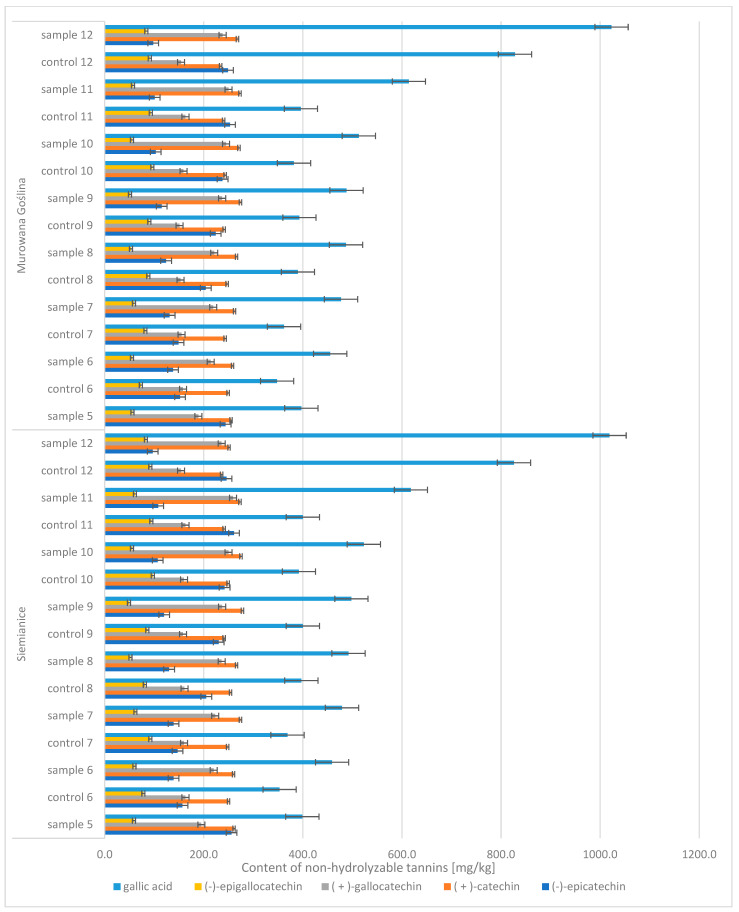
Content of non-hydrolyzable tannins in English oak acorns (Murowana Goślina, Siemianice) (mg/kg); sample, tree with galls; control, healthy tree.

In the case of oak acorns, the phenolic compound profile included diverse groups of compounds, including hydrolyzable tannins and non-tannic hydrolysis products of hydrolyzable tannins (tannic acid, m-digallic acid, ellagic acid, hexahydroxydiphenic acid, (-)-epicatechin-3-gallate) and non-hydrolyzable polyphenols (gallic acid, (−)-epicatechin, (+)-catechin, (+)-gallocatechin, (−)-epigallocatechin) (Figure 5 and Figure 6). It was noticed that the contents of four of these compounds increased with the maturation of acorns, i.e., gallic acid (CONTROL: 348 → 828 mg/kg d.m.; SAMPLE: 397 → 1023 mg/kg d.m.), m-digallic acid (CONTROL: 159 → 262 mg/kg d.m.; SAMPLE: 175 → 327 mg/kg d.m.), ellagic acid (CONTROL: 348 → 630 mg/kg d.m.; SAMPLE: 413 → 771 mg/kg d.m.), and (−)-epicatechin-3-gallate (CONTROL: 32 → 274 mg/kg d.m.; SAMPLE: 30 → 392 mg/kg d.m.).

**Figure 7:**
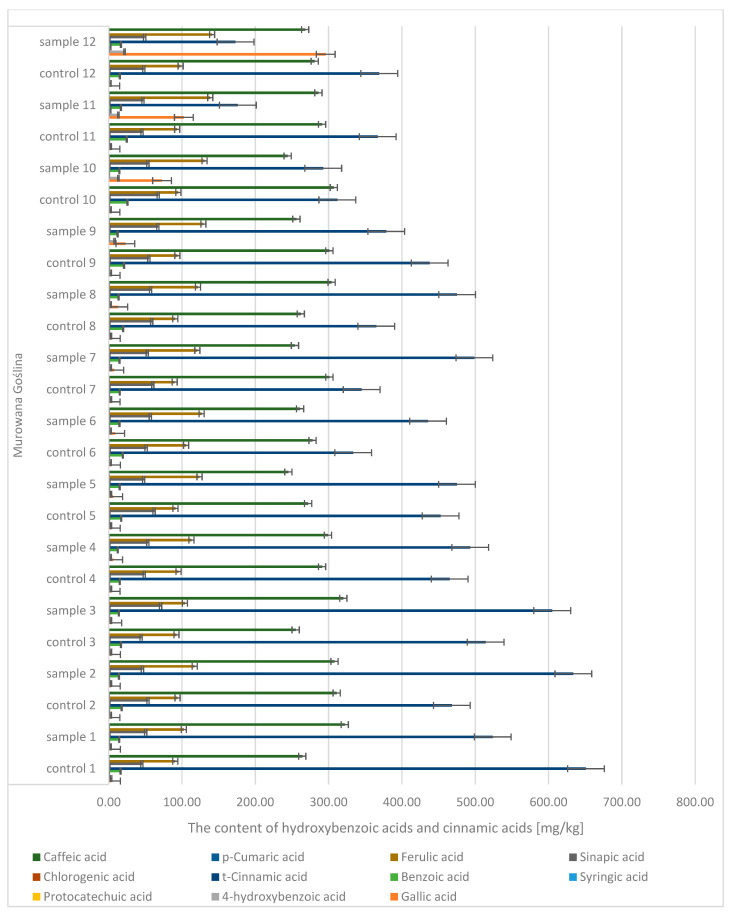
The contents of hydroxybenzoic acids and cinnamic acids in the leaves of the English oak, Murowana Goślina; sample—tree with galls; control—healthy tree.

**Figure 8:**
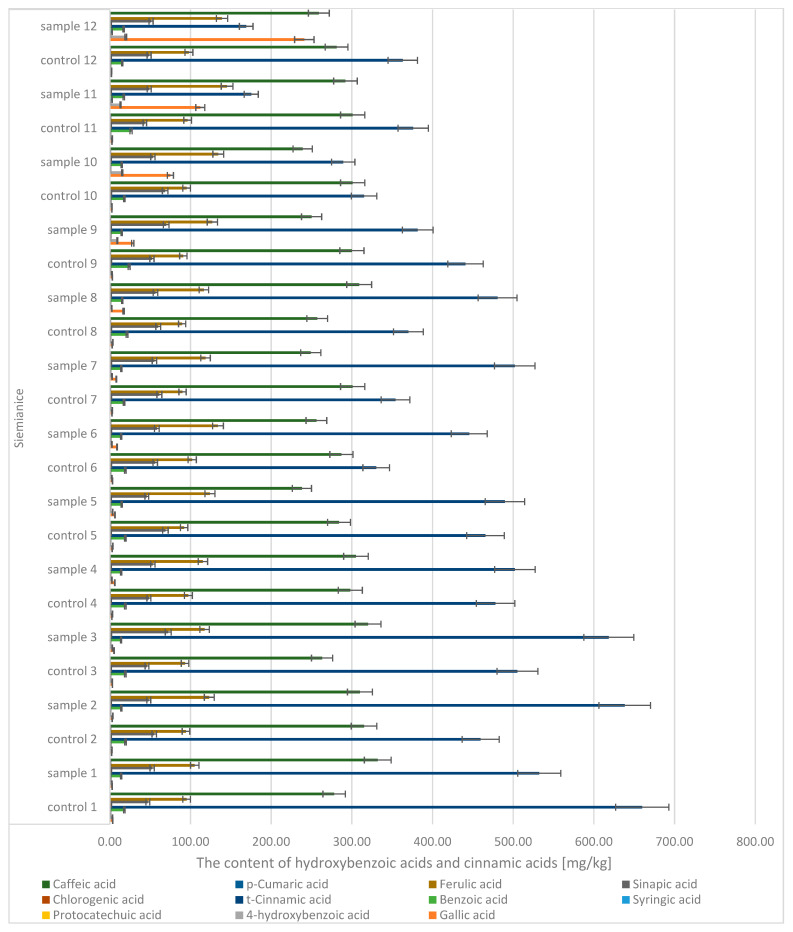
The contents of hydroxybenzoic acids and cinnamic acids in the leaves of the English oak, Siemianice; sample—tree with galls; control—healthy tree.

**Figure 9:**
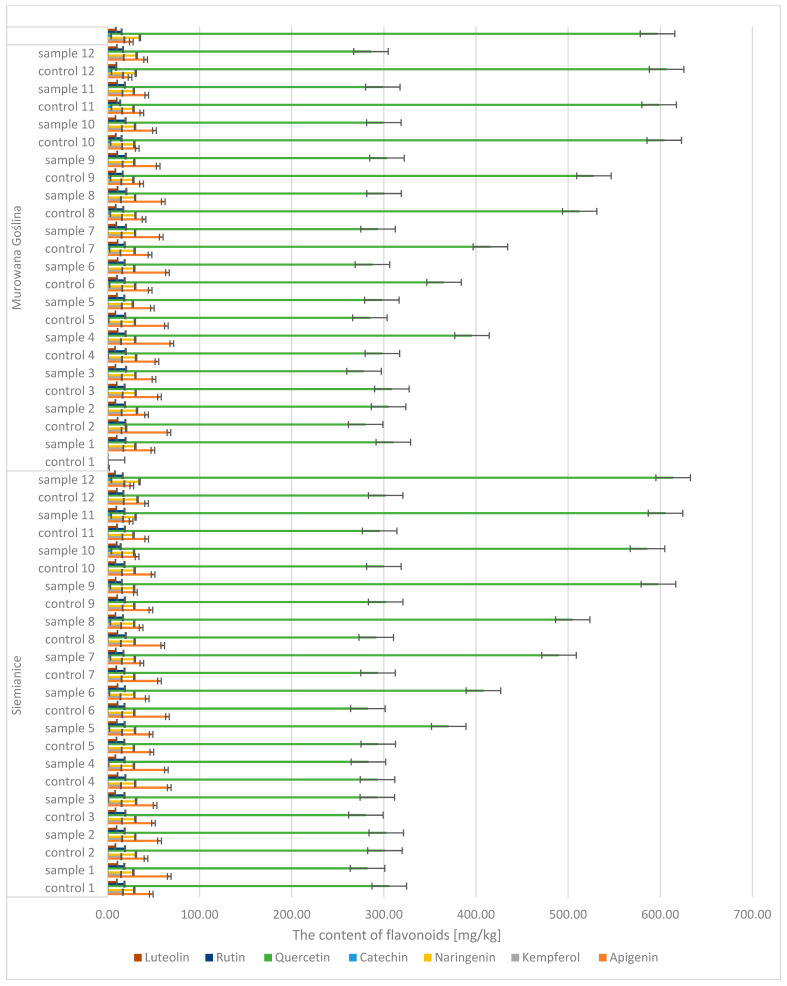
The contents of flavonoids in the leaves of the English oak, Murowana Goślina, Siemianice; sample—tree with galls; control—healthy tree.

Quantitative and qualitative analyses of phenolic compounds in oak leaves revealed the presence of 18 polyphenols, including 11 phenolic acids (gallic, 4-hydroxybenzoic, caffeic, syringic, *p*-coumaric, benzoic, ferulic, sinapic, trans-cinnamic, chlorogenic, and protocatechuic acids) (Figure 7 and Figure 8) and 7 flavonoids (apigenin, catechin, kaempferol, luteolin, naringenin, quercetin, and rutin) (Figure 9). Significant differences were observed between uninfected and infected leaves, with the concentrations of several compounds increasing with the progression of infection. For instance, the gallic acid content ranged from 1.89 to 3.50 mg/kg d.m. in control samples and increased up to 296 mg/kg d.m. in infected leaves. In contrast, in galls, the concentration reached as high as 1589.9 mg/kg d.m., indicating strong accumulation at the infection site. A similar trend was observed for (+)-catechin (0.10–0.16 mg/kg d.m. in control leaves, up to 4.18 mg/kg d.m. in infected leaves, and 58.9 mg/kg d.m. in galls), (−)-epicatechin (22.7 mg/kg d.m. in galls), and (−)-epigallocatechin (251 mg/kg d.m. in galls). High concentrations were also detected for tannic acid (328.6 mg/kg d.m.), ellagic acid (142.5 mg/kg d.m.), and m-digallic acid (101.1 mg/kg d.m.). These findings clearly suggest that gall formation is associated with the intensive biosynthesis and accumulation of phenolic compounds, which may play a crucial role in the plant’s defense mechanisms and in plant–pathogen interactions.

**Figure 10:**
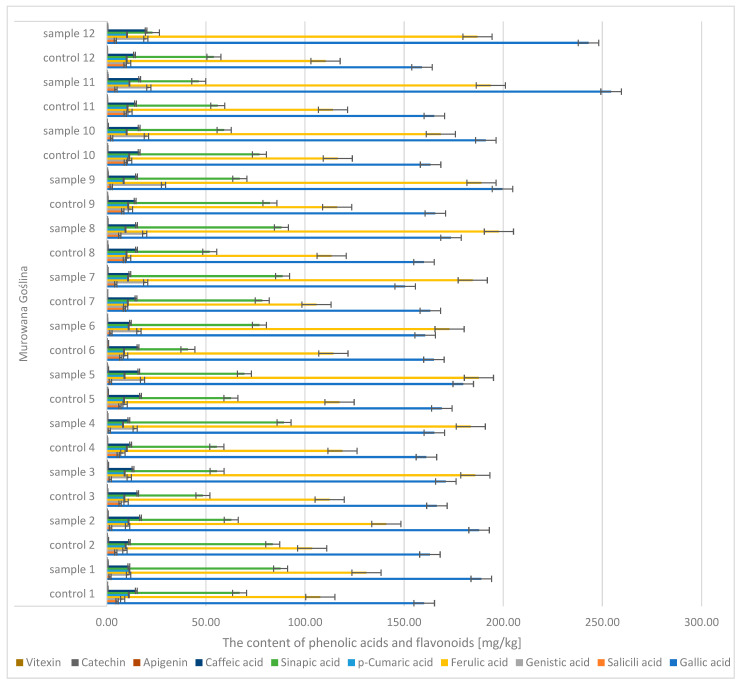
The contents of phenolic acids and flavonoids in the sawdust of the English oak, Murowana Goślina; sample—tree with galls; control—healthy tree.

**Figure 11:**
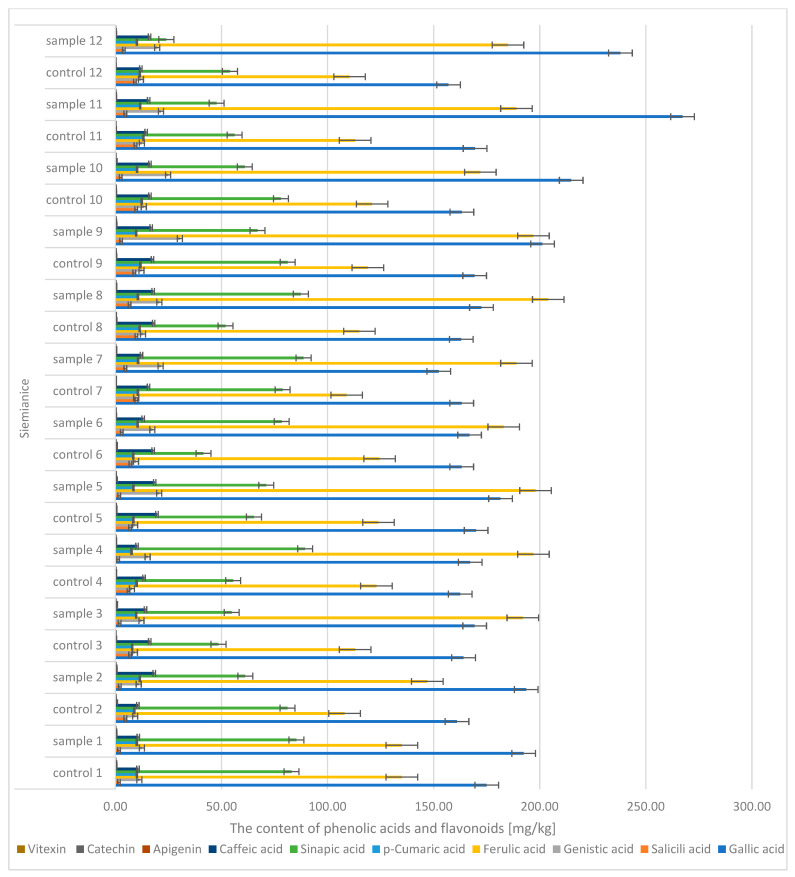
The contents of phenolic acids and flavonoids in the sawdust of the English oak, Siemianice; sample—tree with galls; control—healthy tree.

During the analysis of oak sawdust, the presence of phenolic acids (gallic, caffeic, *p*-coumaric, genistin, salicylic, ferulic, sinapic) and flavonoids (apigenin, catechin, vitexin) (Figure 10 and Figure 11) was determined. In the case of gallic acid, there were significant differences between the control sample (the content was CONTROL: 152 → 170 mg/kg d.m.) and the infected sample (SAMPLE: 150 → 267 mg/kg d.m.). Similar differences were observed in the case of acids: genistin (CONTROL: 8 → 13.4 mg/kg d.m.; SAMPLE: 10.5 → 30.4 mg/kg d.m.) and ferulic (CONTROL: 103 → 124 mg/kg d.m.; SAMPLE: 131 → 204 mg/kg d.m.).

The study showed significant differences in the contents of bioactive compounds between infected and healthy oaks. Although these differences were minimal in flowers, an increase in the contents of some polyphenols, such as gallic acid, ellagic acid, and catechins, was observed in acorns and leaves in infected samples. Similar increases in the contents of bioactive compounds were also observed in the sawdust, where higher concentrations of gallic, genistin, and ferulic acids were observed in infected samples. This confirmed the effect of gall larvae infection on the variability of the profile of bioactive compounds in different parts of the oak.

The contents of bioactive metabolites in galls and their extracts have been the subject of numerous phytochemical studies. In the scientific literature, the total contents of phenolic compounds, flavonoids, and tannins are most frequently determined. Extracts from galls induced by hymenopterans of the family *Cynipidae* have been shown to contain, respectively, the following: 378.73 ± 13.6 mg GAEs/g total phenolics, 108.85 ± 3.37 mg QEs/g flavonoids, and 205.05 ± 5.55 mg CEs/g tannins [23]. Additionally, some studies have identified the presence of specific phenolic compounds, among which the most commonly found were the following: 2,5-dihydroxybenzoic acid, caffeic acid, epicatechin, and ellagic acid. Numerous reports—especially from Asian countries—indicate that galls exhibit a higher diversity of phenolic composition and elevated levels of bioactive metabolites compared to host plant tissues [19,20,21,22]. It has been noted that cynipid galls exhibit greater diversity and higher concentrations of bioactive compounds than their host plant. It is also emphasized that the site of gall formation on the plant significantly influences its chemical composition—secondary metabolite synthesis occurs more efficiently in young tissues with more intensive metabolic activity [24,25,26,27,28,29]. The results obtained in the present study confirm these observations. In the analyzed oak galls, very high concentrations of selected phenolic compounds were found, including gallic acid (1589.9 mg/kg dry matter), tannic acid (328.6 mg/kg d.m.), ellagic acid (142.5 mg/kg d.m.), and m-digallic acid (101.1 mg/kg d.m.). These compounds occurred in galls at concentrations much higher than in uninfected oak leaves, where the gallic acid content did not exceed 3.50 mg/kg d.m. For example, the contents of (+)-catechin and (−)-epicatechin in galls were 58.9 and 22.7 mg/kg d.m., respectively, while in control samples, these values ranged from 0.10 to 0.16 mg/kg d.m. (catechin), and (−)-epicatechin was not detected. Moreover, high concentrations of (−)-epigallocatechin (251 mg/kg d.m.) and (−)-epicatechin 3-gallate (301 mg/kg d.m.) were recorded in galls, whereas these compounds were absent or present only in trace amounts in control leaves. The gathered data clearly indicate that gall formation induces the intense biosynthesis and accumulation of phenolic compounds, which may play a significant role in plant defense mechanisms and plant–pathogen interactions.

Gall induction, development, and interactions with host plants remain poorly understood, despite progress in research on this phenomenon. Galls, resulting from insect activity are a research subject, particularly their influence on the host plant’s anatomy, ultrastructure, and physiological processes [30,31]. To date, gall structures and ultrastructures have been described in the scientific literature, and several studies have been undertaken to understand the mechanisms of their induction [31,32,33,34,35,36,37]. Galls induced by insects from the family *Cynipididae* produce characteristic deformations on oak leaves (*Quercus robur* L.) [38]. Compared to uninfected oak leaves, the anatomy and structure of galls show several significant differences. Different cell layers have been identified, which are formed due to the action of pheromones secreted by insect larvae [38]. As a result of the conducted analyses, it was discovered that the type of storage material in the nutritive tissue of the gall may differ depending on the insect species and its stage of development [23]. In addition, an important finding was that galls of the same insect species can exhibit different structural features depending on the developmental stage of the host plant. This study on the effect of the insect *C. quercusfolii* L. on the oak leaf on which it lays eggs is a breakthrough in this field. In this study, we analyzed the effect of this insect on the level of oxidative stress, which was expressed based on the cation radical ABTS, and the content of biofunctional metabolites. The metabolite content depends on the oxidative stress level, which was measured in key anatomical parts of the oak, with and without galls, throughout the growing season. Based on this study, it was noted that the presence of the insect *C. quercusfolii* L. on the oak leaf affects not only the leaf on which the gall is located but also the entire host plant, starting from flowers during the flowering period, through the full growing season, until the acorn drop. The results of these studies may provide new information on both the local and general effects resulting from the presence of insects in the plant. This study reveals that galls induced by *Cynips quercusfolii* on *Quercus robur* may trigger a systemic biochemical response, not limited to the site of infection. Using UHPLC profiling and antioxidant (ABTS) analysis across multiple organs and time points, the research shows seasonal changes in the accumulation of phenolic compounds, flavonoids, and tannins. These preliminary results suggest that galls may enhance the tree’s overall defense capacity, potentially activating mechanisms such as systemic acquired resistance (SAR). The findings contribute to understanding plant–insect interactions and highlight galls as a possible ecological or pharmacological resource. This study presents a novel approach to analyzing the influence of *Cynips quercusfolii* L. galls on the systemic defense response of *Quercus robur* L. Although oak galls are traditionally considered localized parasitic structures, recent evidence suggests that they may elicit broader physiological responses in the host. From a biological perspective, the gall represents a plant-derived organ integrated with host tissues. The metabolic activity around galls is characterized by the localized accumulation of secondary metabolites, such as tannins, phenolic acids, and flavonoids—compounds with known defensive functions. These include anti-herbivore and antifungal properties and roles in modulating stress tolerance. Our results indicate that gall induction is associated not only with localized changes in leaf chemistry but also with biochemical alterations in distant tissues, suggesting a systemic signaling response. This aligns with the concept of systemic acquired resistance (SAR), in which a local biotic stress triggers defense priming throughout the plant [22,39]. Furthermore, it has been proposed that galls may indirectly reduce herbivore pressure by attracting predators or parasitoids of the gall-inducing insect [20]. In this context, gall formation may play a dual role—both as a cost of parasitism and as a potential contributor to the plant’s integrated defense network.

Although the present study did not include a targeted experimental design to assess proximity effects, field observations revealed some suggestive trends. In several cases, leaves and acorns located close to gall-affected tissues on the same branch exhibited elevated levels of phenolic compounds and antioxidant activity compared to tissues more distant from the gall. This observation may indicate the localized but systemic activation of defense pathways. Such effects are consistent with the concept of systemic acquired resistance (SAR), in which local biotic stress—such as insect galling—can induce signaling cascades affecting distant plant organs [40]. Moreover, studies have shown that the production of flavonoids, tannins, and phenolic acids can be upregulated not only at the site of herbivory behavior but also in uninoculated parts of the same plant [39]. Interestingly, no consistent increase in metabolite contents was observed in uninfected oaks growing near infected individuals, which may suggest that the defense response remains largely individual and internal rather than mediated by interplant signaling (e.g., via volatiles or shared mycorrhizal networks). However, we acknowledge that this hypothesis requires a controlled spatial analysis and metabolic mapping, which may be addressed in future studies.

### 2.1. Relevance and Contribution of the Study

This study provides novel insights into how *C. quercusfolii*-induced galls influence systemic physiological and biochemical responses in *Quercus robur*. While galls have traditionally been viewed as localized parasitic structures, our findings suggest that their presence can trigger broader defense-related metabolic changes across multiple plant organs. By integrating seasonal sampling, antioxidant activity assays (ABTS), and the UHPLC-based profiling of secondary metabolites, this research highlights a potentially systemic plant response to localized biotic stress.

The results demonstrate the following:

- Clear alterations in phenolic profiles and antioxidant activity not only at the gall site but also in distant tissues (sawdust, flowers, acorns);
- A seasonal dynamic in secondary metabolite accumulation likely linked to gall development stages;
- The potential role of galls as indirect defense enhancers.

This contributes to the broader understanding of plant–insect interactions, systemic acquired resistance (SAR), and the ecological function of galls. It also opens new avenues for exploring oak tissues as a source of bioactive compounds with pharmaceutical or ecological applications.

### 2.2. Statistic Analysis

**Figure 12:**
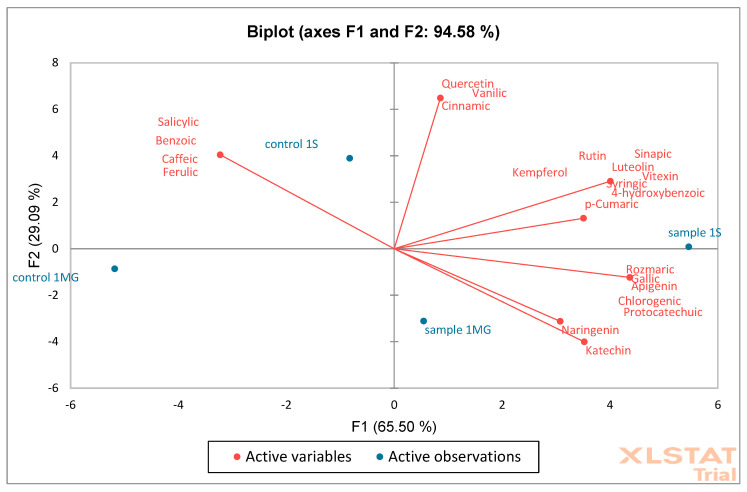
Principal Component Analysis (PCA) was performed on oak flower samples (S—Siemianice, MG—Murowana Goślina). Red arrows represent phenolic compounds, and blue points are samples: sample 1MG, sample 1S—represents experimental samples, control 1MG, control 1S—control samples.

**Figure 13:**
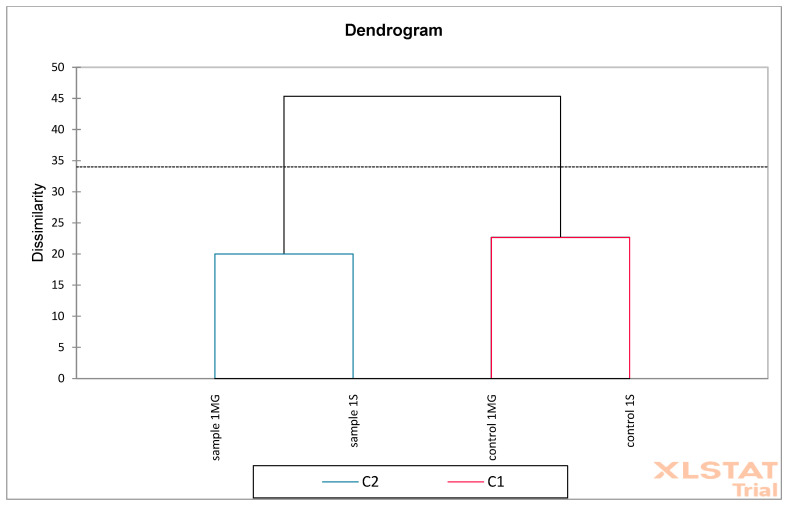
Aggregation Grouping Analysis (AHC) was performed on oak flower samples (S—Siemianice, MG—Murowana Goślina).

Based on the obtained results, PCA (Principal Component Analysis) and AHC (Aggregation Grouping Analysis) were performed on oak flower samples (Figure 12 and Figure 13). The PCA graph presents the analysis of two principal components: F1 (65.50%) and F2 (29.09%). Together, these two components explain 94.58% of the data variability, which indicates very good representativeness of the PCA model. Red arrows represent phenolic compounds, e.g., quercetin, catechin, *p*-cumaric, naringenin, etc. The direction and length of the arrows show the strength and direction of the influence of a given variable on the F1 and F2 components. Variables such as catechin and naringenin strongly correlate positively with the F1 axis. Quercetin and kaempferol have a large contribution to F2. Blue points are samples: sample 1MG, sample 1S—represents experimental samples, control 1MG, control 1S—control samples. Sample 1S and sample 1MG are located on the right side of the F1 axis—they show higher levels of compounds such as catechin and naringenin. Samples control 1S and control 1MG are located more on the left side—they have higher associations with other phenolic compounds, e.g., syringic acid and salicylic acid. A clear separation of control and experimental samples was observed, which may indicate a difference in the phenolic composition.

The quantitative data obtained from HPLC assays were used for Principal Component Analysis (PCA) and hierarchical cluster analysis (AHC). The analyzed variables included concentrations of phenolic compounds, including tannins, flavonoids, phenolic acids, and hydroxycinnamic acids, measured in all samples (flowers, leaves, sawdust, and acorns) for both groups (CONTROL and SAMPLE). The input data for PCA were transformed using z-score standardization, performed separately for each organ type, to minimize the influence of absolute concentration differences between different plant tissues. This standardization allowed for focusing on relative patterns of variability and increased the efficiency of differentiating samples between groups. In cases where the concentration of a given compound was below the limit of quantification (LOD), a value of 0 was assigned. Deficiencies resulting from technical errors or incomplete determinations were supplemented using the k-nearest neighbors imputation method (k = 5). PCA was performed using the XLSTAT package https://www.xlstat.com/ (25 May 2025). A two-dimensional biplot was used to present the results, combining a graph of samples (points) and variables (vectors with points).

For the cluster analysis, the agglomeration method associated with the Euclidean distance metric and the Ward.D2 linking method was used. The optimal cut-off level for identifying two main clusters was determined based on the analysis of the “elbow” plot and the Silhouette coefficient. This level is marked with a dashed line in the dendrograms.

**Figure 14:**
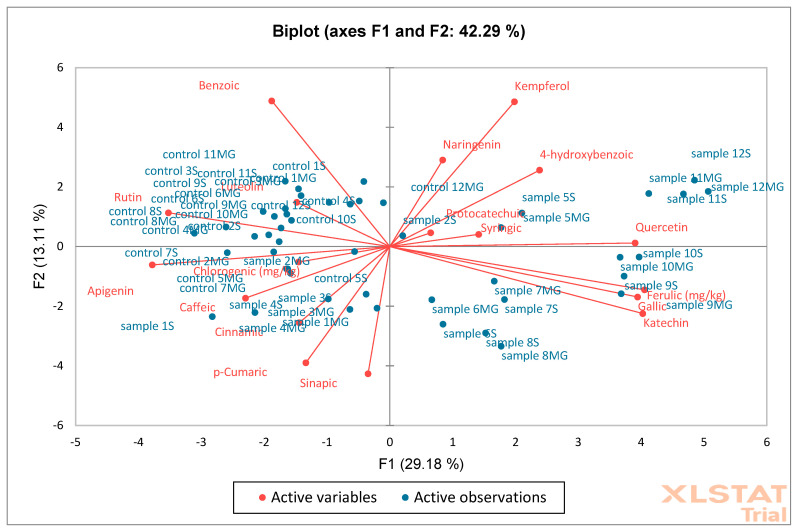
Principal Component Analysis (PCA) was performed on oak leaf samples (S—Siemianice, MG—Murowana Goślina). Red arrows represent phenolic compounds, and blue points are samples: sample 1MG, sample 1S—represents experimental samples, control 1MG, control 1S—control samples.

**Figure 15:**
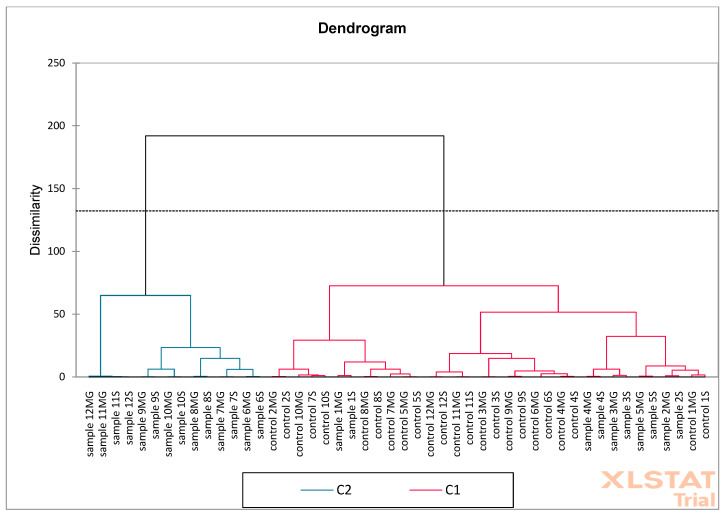
Aggregation Grouping Analysis (AHC) was performed on oak leaf samples (S—Siemianice, MG—Murowana Goślina).

Then, PCA (Figure 14) and AHC (Figure 15) analysis were performed on oak leaf samples. The obtained results showed that the F1 (29.18%) and F2 (13.11%) axes explain 42.29% of the total variance of the data. Red arrows represent active variables (e.g., phenolic compounds such as benzoic acid, kaempferol, catechin, etc.). Blue points are samples labeled as “sample” and “control” with different designations (e.g., 1S, 12MG). Compounds such as catechin, quercetin, 4-hydroxybenzoic acid, and kaempferol are strongly correlated with the F1 axis and separate the samples to the right. Samples located closer to a given variable vector (e.g., “sample 12S” for kaempferol) probably have a higher concentration of this substance. Control samples are mainly concentrated in the center of the system, suggesting their similarity and lack of strong distinguishing features within the variables. Samples labeled as “sample MG” and “sample S” are distributed more broadly, indicating greater chemical diversity. PCA and AHC together indicate that the experimental samples (sample S, MG) are more diverse than the controls, which show relative homogeneity. Samples from the MG and S groups are characterized by a higher proportion of some phenolic compounds (e.g., kaempferol, quercetin). PCA suggests which compounds are responsible for the differences (e.g., kaempferol, catechin), and AHC indicates a clear clustering of samples.

**Figure 16:**
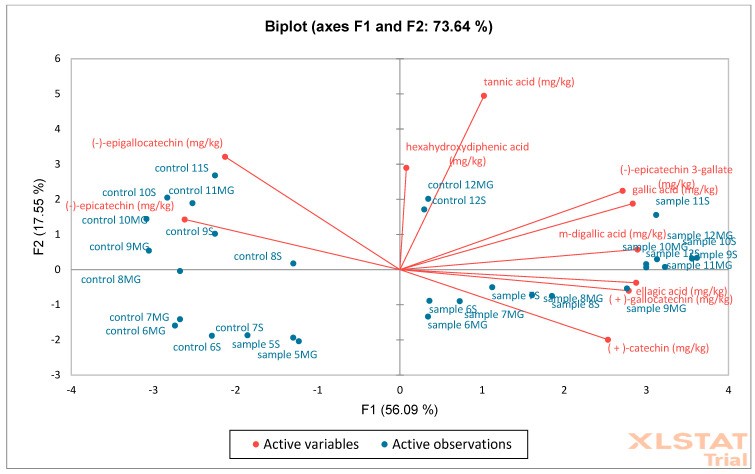
Principal Component Analysis (PCA) was performed on oak acorn samples (S—Siemianice, MG—Murowana Goślina). Red arrows represent phenolic compounds, and blue points are samples: sample 1MG, sample 1S—represents experimental samples, control 1MG, control 1S—control samples.

**Figure 17:**
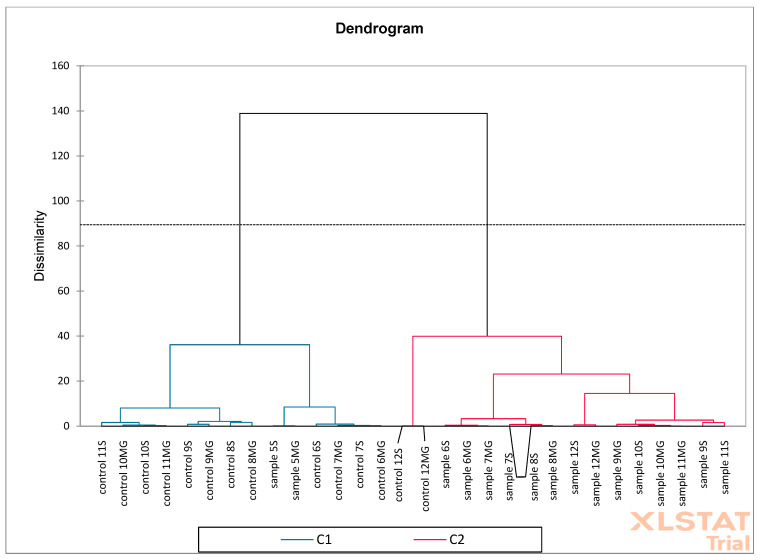
Aggregation Grouping Analysis (AHC) was performed on oak acorn samples (S—Siemianice, MG—Murowana Goślina).

The next analysis performed was PCA (Figure 16) and AHC (Figure 17) on oak acorn samples. Based on the obtained results, it was found that the F1 (56.09%) and F2 (17.55%) axes together explain 73.64% of the total variability in the data, which is a relatively high value. Red arrows represent active variables (e.g., content of phenolic compounds such as catechins, tannic acid, ellagic acid, gallic acid, etc.). Blue points are active observations—samples marked as “control” and “sample” with numbers. Control samples are concentrated mainly on the left side of the F1 axis, which means that they have low values for variables related to the contents of phenolic compounds. Samples are mainly distributed on the right side—they show higher values of compounds such as the following: (+)-catechin, m-digallic acid, gallic acid, (−)-epicatechin gallate, etc. AHC analysis shows a fixed cut-off level (dotted line), which indicates two main groups (clusters): C1 (blue)—contains mainly control samples and several samples (with galls) with similar chemical profiles, C2 (pink)—contains mainly samples (with galls), which differ significantly from the control, suggesting that they have been modified or have a different phenolic composition. Control samples are more homogeneous—they cluster together in compact groups at a low dissimilarity value. Samples (with galls) form a separate cluster, indicating significant differences in the phenolic content (also confirmed via PCA). PCA shows that samples (with galls) differ from “control” mainly due to higher concentrations of some phenolics. AHC confirms this difference by dividing the samples into two main groups: control and test. Both analyses show a clear separation between control and modified/test samples.

**Figure 18:**
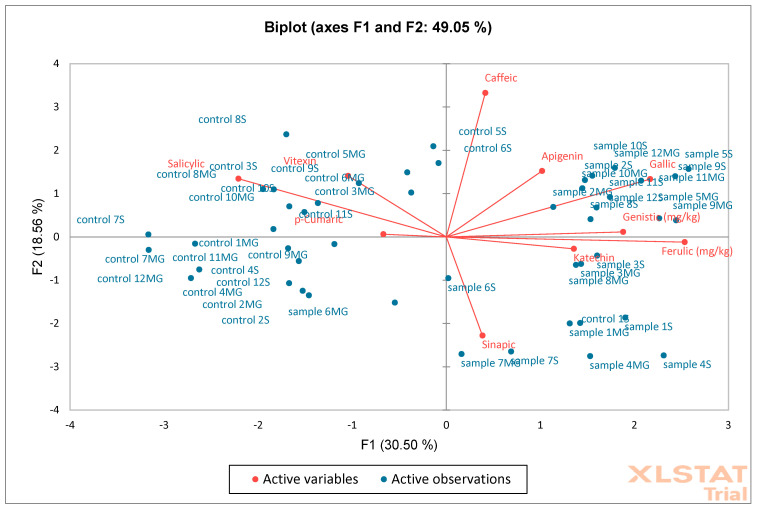
Principal Component Analysis (PCA) was performed on oak sawdust samples (S—Siemianice, MG—Murowana Goślina). Red arrows represent phenolic compounds, and blue points are samples: sample 1MG, sample 1S—represents experimental samples, control 1MG, control 1S—control samples.

**Figure 19:**
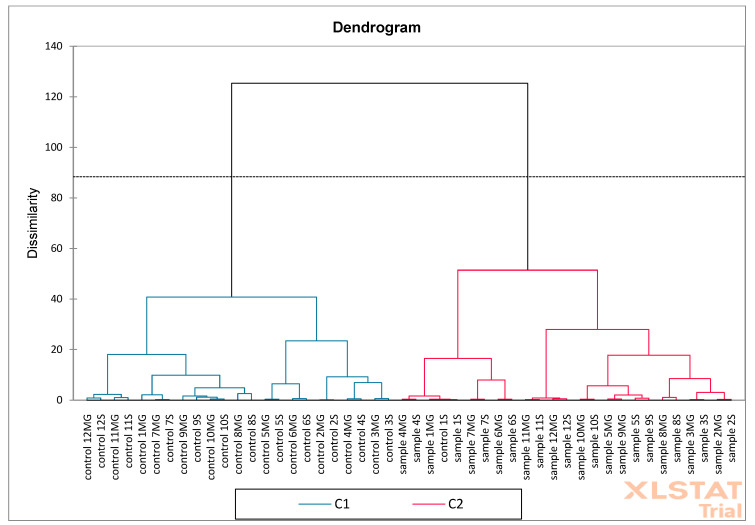
Aggregation Grouping Analysis (AHC) was performed on oak sawdust samples (S—Siemianice, MG—Murowana Goślina).

The last analysis performed was PCA (Figure 18) and AHC (Figure 19) on oak sawdust samples. The results showed that the F1 (30.50%) and F2 (18.55%) axes together explain 49.05% of the total variability in the data. The F1 axis reflects the largest differences between samples and controls. The F2 axis shows additional variability, but less than F1. Most of the controls are on the left side of the graph (F1 negative), while the samples are on the positive side, suggesting significant compositional differences between groups. The phenolic compounds vectors (e.g., caffeic, apigenin, genistic) indicate which compounds influence the discrimination between groups. Compounds such as ferulic, genistic, catechin, apigenin, and caffeic are more associated with samples than with controls (directed towards “sample”). The AHC analysis showed a clear separation of two main clusters indicating significant differences between samples and controls. PCA confirms that the phenolic composition distinguishes samples from controls, with key compounds including ferulic, caffeic, and catechin. AHC shows that control and samples form two distinct clusters, confirming the consistency of the PCA results. Together, both analyses indicate a significant difference between controls and samples, which may suggest an effect of the factor/intervention being studied on the phenolic composition.

## 3. Materials and Methods

In this study, the raw material examined was flowers, acorns, leaves, and sawdust of English oak infected and uninfected with oak gall moth larvae from flowering to full fruit ripeness (i.e., 12 dates × 2 locations × 4 morphological parts × 3 repetitions × 2 variants (I: with galls, II: control) = 576 samples for analysis). The material for the study was collected from the same sites in two locations, i.e., Forest Experimental Plants Murowana Goślina Puszcza Zielonka, 52° 33 17.521″ N 17° 6 32.355″ E, Murowana Goślina, Forest Experimental Plants Siemianice (51° 10 49.322″ N 18° 8 28.383″ E, Siemianice), with 3 replicates from each location, allowing for a comprehensive assessment of the context of oxidative stress. The collected samples were dried and then subjected to chemical analysis, considering the level of antioxidant activity ABTS^+^ and the content of phenolic acids and flavonoids using UPLC with a PDA detector.

The following analytical standards were used for the quantification of phenolic acids, flavonoids, and tannins using UHPLC-DAD:

Tannins and catechins:

- Tannic acid (≥98%)—Sigma-Aldrich, St. Louis, MO, USA
- Gallic acid (≥99%)—Sigma-Aldrich, St. Louis, MO, USA
- m-Digallic acid (≥98%)—PhytoLab GmbH & Co. KG, Vestenbergsgreuth, Germany
- Ellagic acid (≥95%)—Sigma-Aldrich, St. Louis, MO, USA
- Hexahydroxydiphenic acid (≥95%)—PhytoLab GmbH, Germany
- (–)-Epicatechin (≥98%)—Sigma-Aldrich, USA
- (+)-Catechin (≥98%)—Sigma-Aldrich, USA
- (+)-Gallocatechin (≥95%)—Extrasynthese, Genay, France
- (–)-Epigallocatechin (≥95%)—Sigma-Aldrich, USA
- (–)-Epicatechin 3-gallate (≥95%)—Extrasynthese, France.

Flavonoids:

- Apigenin (≥97%)—Sigma-Aldrich, USA
- Catechin (mixture of enantiomers)—Sigma-Aldrich, USA
- Luteolin (≥98%)—Sigma-Aldrich, USA
- Quercetin (≥98%)—Sigma-Aldrich, USA
- Kaempferol (≥98%)—Sigma-Aldrich, USA
- Rutin (≥95%)—Carl Roth GmbH + Co. KG, Karlsruhe, Germany
- Naringenin (≥98%)—Sigma-Aldrich, USA
- Vitexin (≥98%)—PhytoLab GmbH, Germany.

Phenolic acids and derivatives:

- Gallic acid (≥99%)—Sigma-Aldrich, USA
- 4-Hydroxybenzoic acid (≥99%)—Sigma-Aldrich, USA
- Protocatechuic acid (≥98%)—Sigma-Aldrich, USA
- Vanillic acid (≥97%)—Sigma-Aldrich, USA
- Syringic acid (≥97%)—Sigma-Aldrich, USA
- Benzoic acid (≥99.5%)—Sigma-Aldrich, USA
- Salicylic acid (≥99%)—Sigma-Aldrich, USA.

Hydroxycinnamic acids:

- Caffeic acid (≥98%)—Sigma-Aldrich, USA
- *p*-Coumaric acid (≥98%)—Sigma-Aldrich, USA
- Ferulic acid (≥99%)—Sigma-Aldrich, USA
- Sinapic acid (≥98%)—Sigma-Aldrich, USA
- Chlorogenic acid (≥95%)—Sigma-Aldrich, USA
- trans-Cinnamic acid (≥98%)—Sigma-Aldrich, USA
- Rosmarinic acid (≥98%)—PhytoLab GmbH, Germany.

All standards were of analytical grade and were dissolved in methanol or methanol–water (*v*/*v*) mixture prior to use. Calibration curves were constructed using 5–7 concentration levels for each compound, with R^2^ values > 0.99.

In our study, the plant material was classified as a sample when at least 5 fully developed galls of Cynips quercusfolii were visibly present on a single leaf or a group of leaves from the same branch. The selection was based on clear morphological identification of the galls (spherical, up to 10 mm, located on the upper leaf surface), confirmed for consistency across all collected individuals. To reduce the intra-group variability, only tissues with active galls at similar developmental stages were included. In situations where galls were smaller or less numerous, the sample was excluded to ensure uniformity and comparability with control samples.

Regarding the importance of confirming the identity of the gall-inducing species, in our study, the identification of *C. quercusfolii* as the causal agent of the observed galls was based on a combination of morphological characteristics of the galls and the emergence of adult wasps under controlled laboratory conditions. The galls were spherical, smooth-surfaced, and located on the underside of oak leaves (*Quercus robur*), consistent with the typical morphology of *C. quercusfolii* galls, as described in the literature [41]. Mature galls were collected and maintained in aerated containers under ambient conditions until adult gall wasps emerged. The adult wasps were then examined under a stereomicroscope, and identification was confirmed based on taxonomic keys [42], focusing on diagnostic features such as the wing venation, antennal structure, and body coloration. No other gall morphotypes or insect species emerged from the collected samples, reinforcing the species-specific attribution.

The research material was dried in a laboratory oven at 40 °C until a constant weight was achieved. It was then ground using a laboratory mill, and the resulting powder was sieved through a 0.5 mm mesh to obtain a homogeneous, free-flowing material with a uniform particle size distribution.

### 3.1. ABTS^+^ Radical-Scavenging Capacity

The spectrophotometric analysis of the ABTS^+^ radical-scavenging capacity was determined according to the method of Przybylska-Balcerek et al. [43]. ABTS^+^ was produced by reacting 2 mM ABTS^+^ (chemical purity for analytical purposes (CPAP), Sigma-Aldrich, St. Louis, MO, USA) in H_2_O (deionized water) with 2.45 mM K_2_S_2_O_8_ (CPAP, Sigma-Aldrich), and it was stored for 12 h at room temperature in the dark. Approximately 0.5 g of dried and finely ground gall tissue was extracted using 10 mL of 80% methanol (*v*/*v*) in water. The mixture was vortexed (10,000× *g*; 2 min, 4 °C) and then subjected to ultrasonic-assisted extraction in a water bath (40 kHz) at 40 °C for 30 min. After extraction, samples were centrifuged at 10,000 rpm for 10 min, and the supernatant was collected and stored at −20 °C until analysis. The positive control, TROLOX (6-hydroxy-2,5,7,8-tetramethylchroman-2-carboxylic acid), was used at concentrations ranging from 50 to 500 µM to generate the standard curve. The negative control, 80% methanol (the extraction solvent), was used as the blank. ABTS radical-scavenging activity was expressed as µmol TROLOX equivalents (TE) per gram of dry weight (µmol TE/g DW), based on the calibration curve (R^2^ > 0.99). The ABTS^+^ solution was diluted to an absorbance of 0.750 ± 0.025 at 734 nm in 0.1 M sodium phosphate buffer (CPAP, pH 7.4). Then, 1 mL of the ABTS^+^ solution was added to 3 mL of the sample extracts at 100 μg/mL concentrations. The absorbance was recorded for 0.5 h at 734 nm. The extent of decolorization was calculated as a percentage reduction in absorbance.

### 3.2. Extraction of Phenolic Compounds

The samples for the analyses were weighed to 0.20 g. They were placed in sealed 17 mL culture test tubes with a threaded stopper, where alkaline and acid hydrolysis were first run. To run the alkaline hydrolysis, 1 mL of distilled water and 4 mL of 2 M aqueous sodium hydroxide (CPAP, Sigma-Aldrich, St. Louis,, MO, USA) were added to the test tubes. Tightly sealed test tubes were heated in a water bath at 95 °C for 30 min. After cooling (approx. 20 min, to room temperature, approx. 21 °C), the test tubes were neutralized with 2 mL of a 6 M aqueous hydrochloric acid solution (pH = 2) (CPAP, Sigma-Aldrich). Next, the samples were cooled in water with ice. The flavonoids were extracted from the inorganic phase using diethyl ether (2 × 2 mL) (CPAP, Sigma-Aldrich). Formed ether extracts were continuously transferred to 8 mL vials. Next, acid hydrolysis was run. For this purpose, the aqueous phase was supplemented with a 3 mL of a 6 M aqueous hydrochloric acid solution. Tightly sealed test tubes were heated in a water bath at 95 °C for 30 min. After being cooled in water with ice, the samples were extracted with diethyl ether (2 × 2 mL). The produced ether extracts were transferred to 8 mL vials and then evaporated to dryness in a stream of nitrogen (technical gas, purity 5.0—99.999%). Before the analyses, the samples were dissolved in 1 mL of methanol (purity to HPLC, Sigma-Aldrich) [44,45,46].

### 3.3. Chromatographic Analysis

The analysis used an Acquity H class UPLC system equipped with a Waters Acquity PDA detector (Milford, MA, USA). The chromatographic separation was performed on an Acquity UPLC^®^ BEH C18 column (Watersy, Dublin, Ireland) (100 mm × 2.1 mm, particle size 1.7 µm) (Watersy, Dublin, Ireland). The elution was performed in a gradient using the following mobile phase composition: A, acetonitrile with 0.1% formic acid, and B, 1% aqueous formic acid mixture (pH = 2) (purity to HPLC, Sigma-Aldrich) (Table 1). The concentrations of phenolic compounds were determined using an external standard at wavelengths λ = 320 nm and 280 nm and finally given in mg/1 g of extract. The compounds were identified by comparing the retention time of the analyzed peak with the standard retention time, adding a specific amount of standard to the analyzed samples, and repeating the analysis. The detection limit was 1 µg/g [47].

### 3.4. Statistic Analysis

Statistical data analysis was performed using analysis of variance (ANOVA) and Principal Component Analysis (PCA), utilizing the XLSTAT add-on for Microsoft Excel [48]. The analyses were conducted in accordance with established standards for experimental and exploratory data. One-way ANOVA was applied to assess the statistical significance of differences between the means of more than two independent groups. The input data included a dependent variable (measurement values) and a grouping variable (e.g., experimental categories). In XLSTAT, the “One-way ANOVA” option was selected, and the significance level was set at 0.05. Principal Component Analysis (PCA) was used to reduce the dimensionality of the data and to visualize the structure of relationships between variables and observations. Prior to the analysis, data were standardized due to differences in measurement units among variables. The PCA function, available under the “Dimension Reduction” section in XLSTAT, was used. Results were presented using two-dimensional biplots, which allowed for the interpretation of similarities among situation and variables. Both ANOVA and PCA served as fundamental tools for evaluating relationships and the data structure, enabling hypothesis testing and the exploration of complex patterns of variability. Agglomerative Hierarchical Clustering (AHC) was used to identify and group the dataset based on the similarity between observations. Analysis was performed using the AHC function available in the XLSTAT add-in for Microsoft Excel, in the “Clustering” section. Before clustering, the dataset was normalized to eliminate the influence of differences in the scales of the variables. The Euclidean distance was used as a measure of dissimilarity, and Ward’s method was applied for the association, minimizing the total within-cluster variance at each stage of the clustering process. The resulting dendrogram provided a graphical representation of the clustering structure, allowing for the identification of natural groupings between observations.

## 4. Conclusions

Based on the studies conducted, bioactive metabolites, which include phenolic compounds, were found. For the first time, the contents of phenolic acids, flavonoids, and tannins were determined at each stage of the vegetation cycle of the English oak, starting with flowering and ending with the fall of acorns. These compounds were determined not only in healthy trees but also in trees infected with larvae of *C. quercusfolii* L. This work gained value because it was noticed that galls on leaves have an extraordinary effect on the entire plant. It was found that galls affect the content of the aforementioned compounds and thus can play an important protective function of the plant, both against pathogens and phytophages. Understanding this phenomenon allows for a better understanding of the English oak’s defense mechanisms and an assessment of the potential role of galls in protecting trees against pathogens and phytophages.

## Figures and Tables

**Table 1:** Gradient elution for phenolic compounds in UPLC (C18 column; mobile phases: A: acetonitrile with 0.1% formic acid, B: 1% aqueous formic acid solution (pH ~ 2)).

Time (min)	% A (Acetonitryl + 0.1% HCOOH)	% B (1% HCOOH in Water)
0	5	95
10	30	70
20	50	50
25	95	5
27	95	5
28	5	95
30	5	95

## Data Availability

Data are contained within the article and Supplementary Materials.

## Supplementary Materials

The following supporting information can be downloaded at: https://www.mdpi.com/article/10.3390/molecules30132687/s1.
